# Digital workflow to assess gingival recession coverage independently of the cemento-enamel Junction: a prospective clinical study using the modified coronally advanced tunnel technique with porcine dermal matrix

**DOI:** 10.1007/s00784-024-05936-4

**Published:** 2024-10-27

**Authors:** Gerhard Iglhaut, Tobias Fretwurst, Larissa Schulte, Anton Sculean, Kirstin Vach, Katja Nelson, Victoria Constanze Landwehr

**Affiliations:** 1 Department of Oral and Maxillofacial Surgery/Translational Implantology, Center for Dental Medicine, Medical Center, Faculty of Medicine, University of Freiburg, Hugstetter Straße 55, Freiburg, 79106 Germany; 2 Department of Periodontology, School of Dental Medicine, University of Bern, Freiburgstrasse 7, Bern, 3010 Switzerland; 3 Institute of Medical Biometry and Statistics, Faculty of Medicine and Medical Center, University of Freiburg, Stefan-Meier-Straße 26, Freiburg, 79104 Germany

**Keywords:** Coronally advanced tunnel technique, Digital measurement, Gingival recession, Root coverage, Recession type

## Abstract

**Objectives:**

The limited number of studies using digital workflows to measure soft tissue changes depend on the cemento-enamel junction (CEJ), which has been reported to be unreliable. Our primary objective was to apply an advanced digital assessment method, measuring independent from the CEJ to evaluate the modified coronally advanced tunnel technique (MCAT) with a porcine dermal matrix (PDM) for gingival recession coverage.

**Materials and methods:**

Patients with type RT1 and RT2 gingival recessions were treated with the MCAT and a PDM. Plaster casts (preoperative and 6 months postoperative) were digitalized. Subsequent stereolithography (STL)-files were imported and superimposed in the open-source software GOM Inspect for computer-based analysis. Recession depth, mean root and complete root coverage (mRC and cRC), mean recession reduction (mRR) and gingival thickness were evaluated. Statistical analysis was performed using mixed linear models.

**Results:**

A total of 82 teeth (19 patients) were included in the study. Healing was uneventful in all patients. The mean preoperative recession depth was 1.34 ± 0.92 mm. mRC was 65.06 ± 48.26%, cRC was 25.61%, mRR was 0.87 ± 0.83 mm, and gingival thickness gain was 0.33 ± 0.30 mm, with comparable results for RT1 and RT2. Neither tooth type nor type of jaw had any effect on the amount of root coverage.

**Conclusions:**

The digital evaluation workflow employed offers an approach to evaluate gingival recession coverage outcomes independent of the CEJ. The PDM used in combination with the MCAT shows promising results for root coverage.

## Introduction

Half of the global population is affected by soft tissue recessions [1], which are characterized by a displacement of the marginal gingiva in an apical direction beyond the cementum-enamel junction (CEJ) [2]. The presence of a thin gingival phenotype is a predisposing factor for the development and progression of gingival recessions [3]. However, even in patients with a thick gingival phenotype, the presence of highly inserted frenulums [1] or toothbrushing trauma [4] can lead to gingival recessions. Soft tissue recessions are most commonly located on the buccal surfaces [2], with single-rooted teeth being affected more frequently and severely than molars [5]. An increased prevalence of gingival recession is found in males [6], in the mandible and it increases with age [6]. The Recession type (RT) classification is increasingly being used to classify gingival recessions as the clinical discrimination between Miller class (MC) I and II as well as between MC III and IV remains difficult [4, 7].

The treatment of choice is root coverage and soft tissue thickening either by gingival grafting or guided tissue regeneration [1]. Various surgical treatment strategies have been described in the literature. To cover multiple gingival recessions, the modified coronally advanced tunnel technique (MCAT) is an established treatment technique [8, 9]. In this approach, a tunnel is prepared and mobilized beyond the mucogingival border through sulcular incisions while preserving the interdental papillae. In accordance with the bilaminar technique, a tissue graft is inserted and the tunneled tissue, along with the interdental papillae, is displaced coronally to cover the graft completely [10–14]. Autologous connective tissue grafts, combined with bilaminar techniques to ensure blood supply to the graft, provide the most predictable results and are considered the gold standard [15]. However, the use of autologous connective tissue grafts has disadvantages e.g. donor site morbidity [16]. Therefore, matrices have been suggested as an alternative to autologous connective tissue grafts. Matrices serve as a scaffold for endothelial cells and fibroblasts and are available from xenogenic or allogenic origin [17]. By supporting the immigration of epithelial cells, the matrices are replaced by newly formed connective tissue at the recipient site within six to ten months [18, 19]. However, controlled clinical studies evaluating the rate of root coverage in multiple gingival recessions have shown an average underperformance of up to 27% for the xenogeneic dermal matrice compared to autologous connective tissue grafts [10, 16, 20, 21]. The overall rates for mean and complete root coverage in previous randomized controlled trials (RCTs) employing porcine dermal matrices to address Miller class I and II recessions through the modified coronally advanced tunnel technique have been reported in the range of 53–93% (mean root coverage, mRC) and 20–33% (complete rot coverage, cRC) [16, 20, 22].

In addition to the use of different soft tissue grafting materials, surgical techniques, patient characteristics, and variable observation times, the differences in root coverage results are mainly due to the underlying measurement methodology [7, 23]. Clinical measurements of soft tissue changes are commonly performed with a periodontal probe using the CEJ [23]. However, identification of the CEJ is often compromised and has been considered to be an unreliable reference point since the CEJ is in about 60% of the cses clinically indetectable [24, 25]. Both prominent parameter mRC and cRC utilize the CEJ as a reference point, which leads to a significant source of measurement inaccuracies [23].

Therefore, in recent years, digital measurements of soft tissue changes based on 3D superimposition of pre- and postoperative data have been proposed [26]. However, as highlighted critically by Kuralt et al., in the majority of the 8 available clinical studies the established measurement methods are just digitized, thus still relying on the CEJ [27]. Moreover, the limited number of studies employing new digital evaluation workflows demonstrates significant variability, thereby making comparisons difficult.Therefore, the primary objective of this clinical study was to apply an advanced digital assessment, emphasizing independence from the CEJ, using the MCAT technique with a PDM for gingival recession coverage.

## Materials and methods

### Study design and trial registration

The study was designed as a prospective, single-arm clinical study. It was registered at the Deutsches Register Klinischer Studien/German Clinical Trail Register (DRKS, DRKS00023201) and followed the STROBE guidelines (https://www.equator-network.org/reporting-guidelines/strobe/). The study protocol was approved by the ethics committee belonging to the Faculty of Medicine, University of Freiburg, Germany (No 352/19) and is in accordance with the Declaration of Helsinki of 1975, revised in Fortaleza in 2013. All participants were informed and understood the objectives and the details of the study and signed a written informed consent document.

### Participants

Patients were recruited from November 2019 to September 2022 at the Department of Oral and Maxillofacial Surgery, Faculty of Medicine, University of Freiburg, Germany. Patients with single or multiple gingival recessions (RT1 or RT2) in the maxilla and/or the mandible underwent root coverage with a PDM (Novomatrix™ Reconstructive Tissue Matrix; BioHorizons Camlog, Basel, Switzerland) using the modified coronally advanced tunnel technique [8, 9]. All patients reached the age of legal majority and presented with good oral hygiene (full mouth plaque score < 20%).

Gingival recessions classified as MC IV or RT 3, periodontitis, systemic disorders (such as diabetes, immunosuppression, cardiovascular diseases, irradiation and chemotherapy), disturbed or altered bone metabolism (such as osteoporosis, hormone supplementation and antiresorptive therapy) and parafunctional habits were exclusion criteria. Nicotine users and patients participating in other studies were excluded in the present study.

### Intervention

**Fig. 1:**
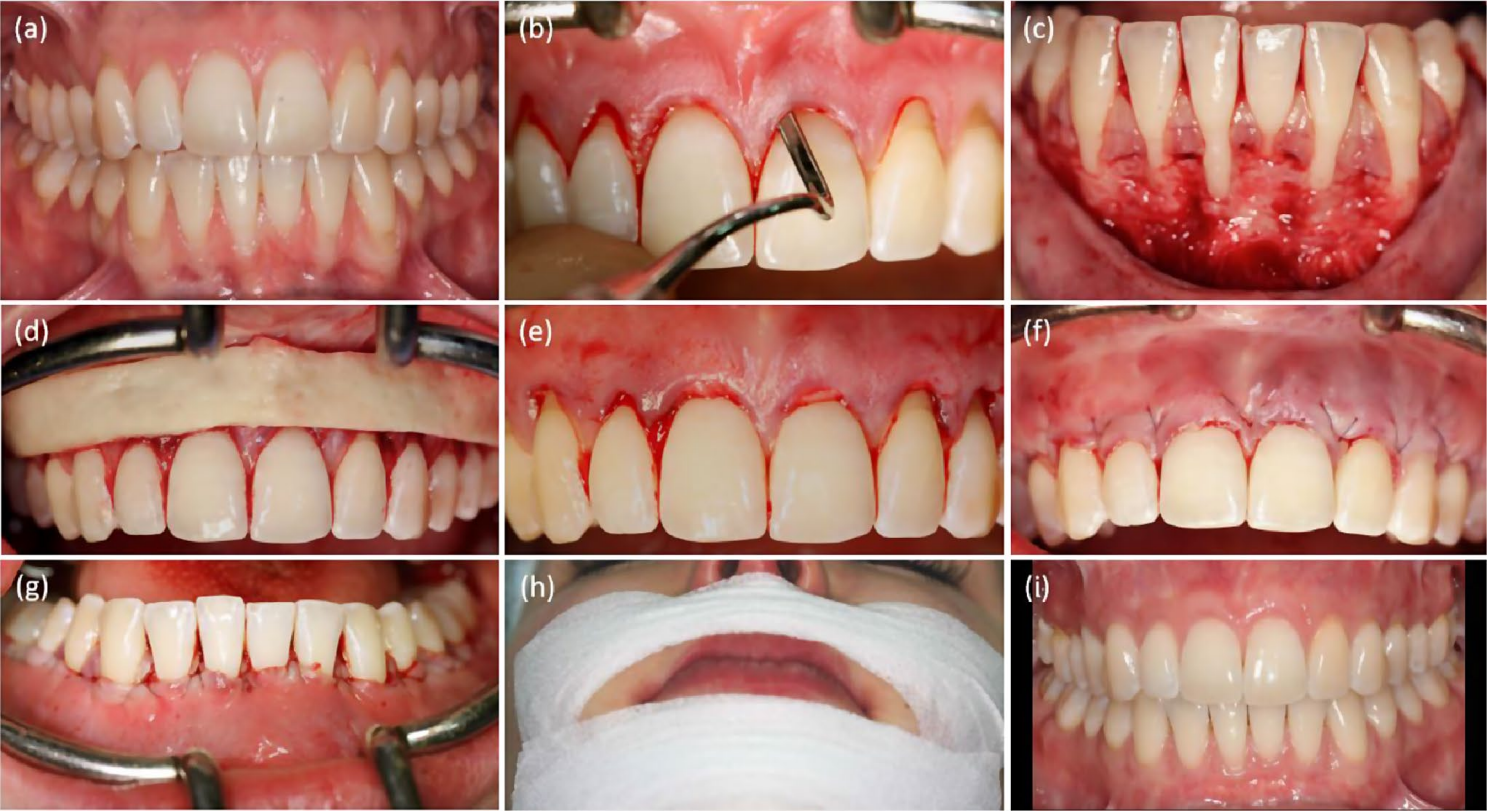
Root coverage with porcine dermal matrix (PDM) combined with the modified coronally advanced tunnel technique (MCAT). (**a**) Preoperative situation (**b**) Preparation of a continuous subperiosteal tunnel (**c**) Subperiosteal intraoperative situation (**d**) Adjustment of the PDM (**e**) Application of the PDM (**f**) Fixation of the PDM with loop sutures in the maxilla(**g**) Fixation of the PDM with loop sutures in the mandible (**h**) Application of an extraoral tape (**i**) Clinical results 6 months postoperatively

Root coverage procedure was performed by a single experienced surgeon (GI). Each patient received professional tooth cleaning and alginat casts (Pluralgin NF, Pluradent GmbH & Co. KG, Offenbach am Main, Germany) three to five days preoperatively. Starting on the evening before surgery, oral antibiotic therapy (amoxycycline 1000 mg three times a day) was initiated and continued for 10 days. Immediately prior to surgery, patients received 600 mg of ibuprofen. Venous blood was drawn for the collection of leukocyte- and platelet-rich fibrin (L-PRF^®^, IntraSpin^®^, Intra-Lock, Boca Raton, FL, USA). Local anesthesia (Artinestol^®^ 1:200,000, Merz Dental, Lütjenburg, Germany) was applied. Cleaning and smoothing of the exposed root surfaces using a curette (Younger-Good curette 7/8, HuFriedy, Chicago, USA) was followed by their conditioning with EDTA gel (PrefGel^®^, Straumann, Basel, Switzerland) for two minutes. Subsequent surgical root coverage using minimally invasive modified coronally advanced tunnel technique was performed (Fig. 1) [28].

The gingiva was detached from the bone surface by a sharp intrasulcular incision using a microblade (Key Dent^®^, American Dental Supply, Vaterstetten, Germany). Tunneling instruments (Iglhaut Tunnel Set, HuFriedy, Chicago, IL, USA) were used to bluntly prepare a continuous subperiosteal tunnel without perforating the flap tissue or detaching the papillae. Only in patients with extremely thin gingival phenotype, a papilla base incision followed by a sharp supraperiosteal preparation was performed due to the increased risk of soft tissue perforation. The PDM was washed in sterile saline for at least five minutes before leukocyte- and platelet-rich fibrin was applied to the matrix. The matrix was then placed by using a curette 7/8 (Younger-Good, Hu-Friedy Group, Frankfurt am Main, Germany) and fixed in the tunnel. The tunnel tissue was displaced coronally to the enamel-cement interface using loop sutures (6 − 0 Seralene^®^, Serag-Wiessner, Naila, Germany). An extraoral tape (Fixomull stretch^®^, BSN medical, Hamburg, Germany) was applied for five days to prevent swelling and mobilization. Postoperatively, all patients were instructed for physical rest and use of an oral rinse (Salviathymol N^®^, Meda Pharma, Radebeul, Germany two times a day) for three weeks. Patients refrained from other oral hygiene measures in the wound area for one week. Clinical evaluation was performed at 1, 10 and 30 days postoperatively to assess complications such as infection, hematoma, postoperative pain, nerve injury, wound dehiscence, duration of analgesic use and overall acceptance of the root coverage procedure. Suture removal was performed four weeks postoperatively. Clinical parameters were assessed at six months postoperatively.

### STL file acquisition and 3D superimposition

**Fig. 2:**
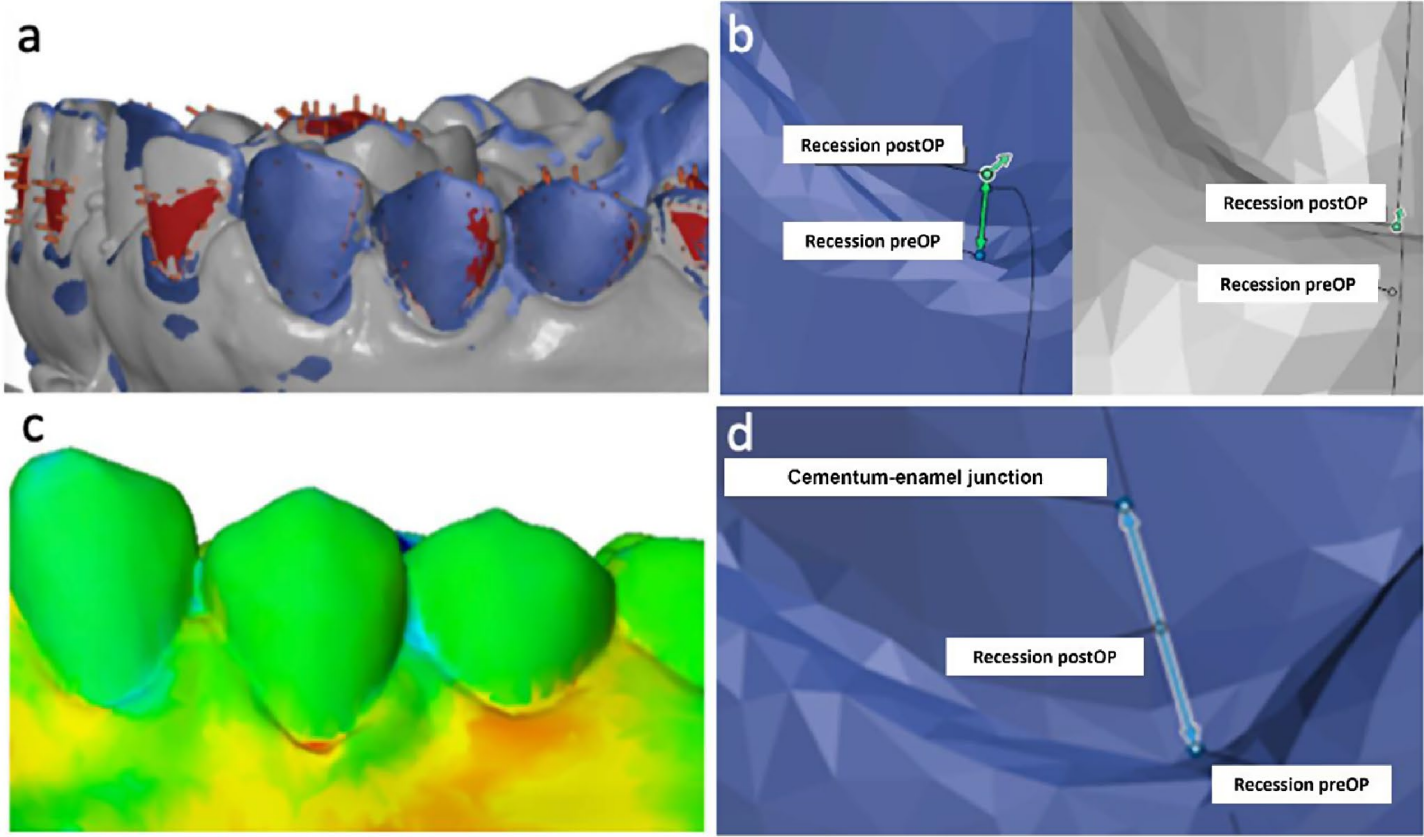
Workflow of the digital recession assessment. (**a**) 3D Superimposition of the pre-/postoperative digitized models (**b**) Measurement of reduction of recession depth by using “Construct 2-Point Distance” function (preoperative recession/postoperative recession) (**c**) Visualization of gingival thickness gain using a heat map showing positive (yellow/orange/red) and negative (blue) changes in gingival thickness (**d**) Mean root and total root coverage analysis. Recession depth was determined as distance between the deepest point of recession and the CEJ along the tooth axis

Plaster casts based on alignate impressions (Pluralgin NF, Pluradent GmbH & Co. KG, Offenbach am Main, Germany) were anonymized and digitized using the 3Shape Lab Scanner E3 (3Shape, Copenhagen, Denmark). The resulting STL files were imported into the open-source software GOM Inspect (Carl Zeiss GOM Metrology GmbH, Braunschweig, Germany) for computer-based analysis. The digitized cast acquired six months postoperatively were imported as a “mesh” and superimposed on the “CAD body” using the full arch to superimpose the preoperative and postoperative scans. The initial alignment was rendered by the software and manually optimized by selecting tooth surfaces (Fig. 2).

### Root coverage assessment

Root coverage assessment (Fig. 2) was performed three times per tooth by two independent investigators (LS; SO). The mean value of the measurements was calculated. The following root coverage parameter were applied:

Parameter dependent on CEJ.

It has been common practice in periodontal research to measure recession coverage using “CEJ-dependent” parameters. To ensure comparability with existing research, the present study used the digitized CEJ in addition to CEJ-independent parameters:

#### Recession depth

The distance between the deepest point of recession and the CEJ along the tooth axis determined the preoperative recession depth.

#### Mean root and total root coverage

Mean root coverage was calculated by dividing the difference between the preoperative and postoperative recession depth by the preoperative recession depth and then multiplying the results by 100 [29]. Complete root coverage was calculated by dividing the number of teeth with postoperative covered roots extending to or beyond the CEJ by the total number of teeth multiplied by 100 [30].

Due to the small color differences, the CEJ could not be detected on the digital models. Therefore, the CEJ was identified on the stone models and transferred to the digital models. If the CEJ was not clearly visible on the plaster cast, the cases were not included in the study.

### Parameter independent of CEJ

As the literature has shown that the CEJ is a problematic reference point, this study introduces a new method for assessing recession coverage independent of the CEJ.

#### Quantification of reduction of recession depth

The reduction of the recession depth was measured in mm between the point of “preoperative recession” and “postoperative recession” using the “Construct 2-Point Distance” function in the GOM Inspect software. The reduction of recession depth parameter is equal to the absolute gain of gingival height described by Xue et al. [31].

#### Gingival thickness

A surface comparison on the “CAD body” was performed. A plane was created through the most caudal recession point per tooth. Values were gathered each 3 mm in coronal and apical direction in 1 mm single steps. The calculated mean value yields the postoperative change of gingival thickness.

### Statistical analysis

The sample size for this single-arm non-inferiority study was planned for the parameter mean root coverage with a comparative value of 87% (as the mean value of the publications by Aroca and colleagues [10, 32] and a non-inferiority margin of 10%. Assuing a standard deviation of 25%, a toal of 66 teeth are required with a power of 90%, an apha of 5% and a assumption of a variance inflation factor of 3 if more than one tooth per patient is considered.

For statistical analysis, mean and standard deviation as well as the intraclass correlation coefficient were calculated. A graphical representation was provided by bar charts and box plots. Linear mixed models with patient as a fixed effect were used to analyze differences in the several outcome parameters regarding jaw, type of tooth and recession type. All subsequent tests carried out in pairs were corrected for multiple testing using the method of Scheffe. All data were analyzed using STATA 17.0 (StataCorp, College Station, Texas USA).

## Results

### Baseline data

A total of 82 teeth (38 maxilla; 44 mandible) in 19 patients (15 females and 4 males; mean age 46.09 ± 14.94 years) with gingival recessions classified as RT1 (*n* = 39) or RT2 (*n* = 43) were included (Table 1).

**Table 1:** Patient and site characteristics at baseline

Parameter	
Patient number (n)	19
Teeth (n)	82
Age (mean ± SD) (years)	46.09 ± 14.94
Gender (Female/Male) (n)	15/4
Gingival recession type (n)	RT1: 39 RT2: 43
Maxillary/mandibular sites (n)	38/44
Maxillary sites (n)	38
Central/lateral incisor sites (n)	7
Canine sites (n)	9
Premolar sites (n)	11
Molar sites (n)	11
Mandibular sites (n)	44
Central/lateral incisor sites (n)	14
Canine sites (n)	7
Premolar sites (n)	15
Molar sites (n)	8
Recession depth (mean ± SD) (mm)	1.34 ± 0.92

Three patients received recession coverage in the maxilla as well as in the mandible. Six of the 25 patients initially enrolled were excluded: Three of these patients received a new prosthetic restoration, precluding a comparison of data, one patient discontinued the study at his own request, one patient being excluded due to lack of compliance, and one patient being excluded due to digital model matching issues.

**Fig. 3:**
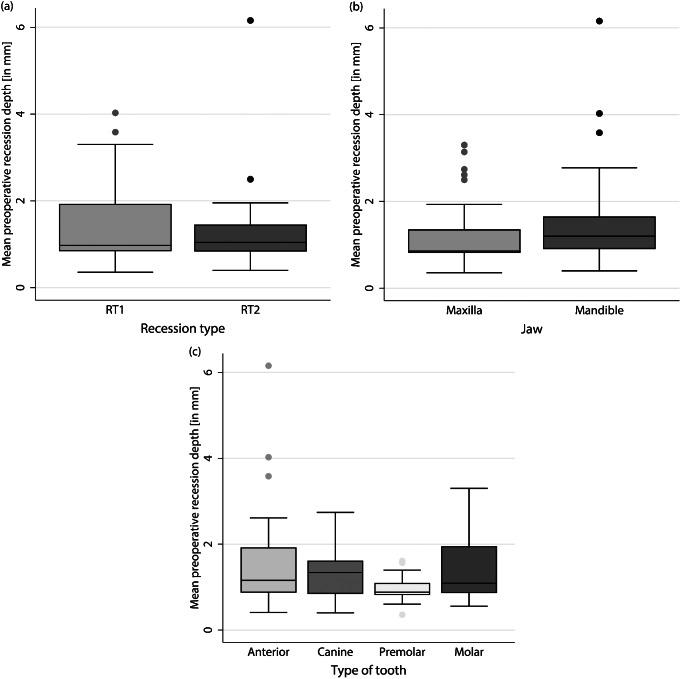
Mean preoperative recession depth was differentiated based on (**a**) Recession type, (**b**) Jaw type and (**c**) Tooth type in mm. Measurement was performed digitally between the most apical point of the gingival margin and the CEJ

The mean preoperative recession depth was 1.34 ± 0.92 mm (RT1: 1.44 ± 0.95 mm; RT2: 1.25 ± 0.89 mm; maxilla: 1.18 ± 0.74 mm; mandible: 1.47 ± 1.03 mm). Differentiated by tooth type, the mean recession depth was 1.70 ± 1.39 mm for anteriors, 1.33 ± 0.66 mm for canines, 0.94 ± 0.30 mm for premolars, and 1.52 ± 0.87 mm for molars (Fig. 3).

### Clinical outcome

Graft healing was uneventful in all patients, there were no postoperative complications such as pain, hematoma formation, infection, nerve injury or wound dehiscence. All enrolled patients showed good esthetic results with no gingival scarring or discoloration in the operation area at six months postoperatively (Fig. 1).

### Mean root coverage

**Fig. 4:**
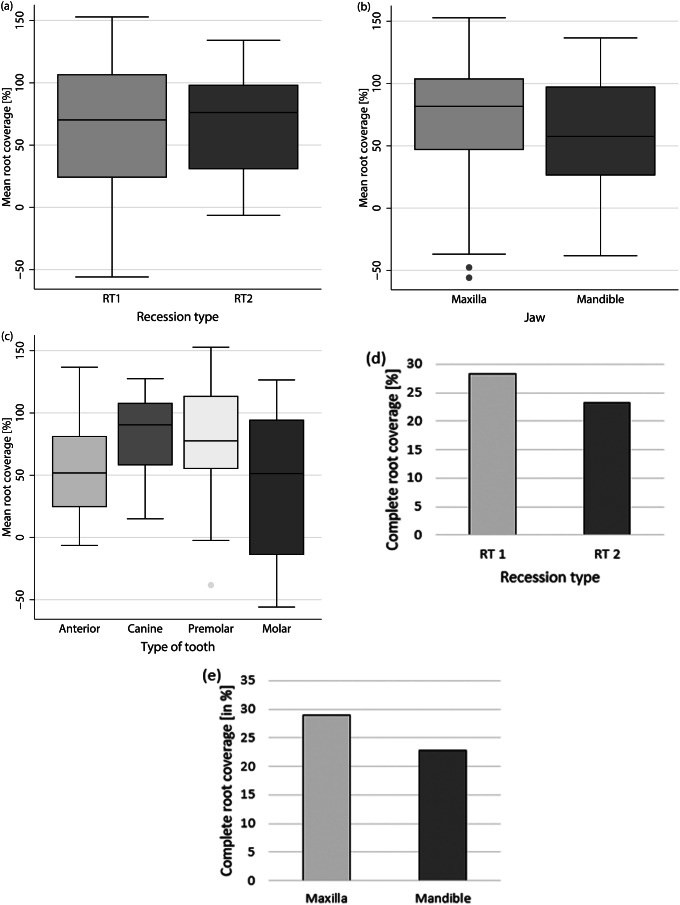
Mean root coverage in % according to (**a**) Recession type, (**b**) Jaw type, and (**c**) Tooth type and complete root coverage in % according to (**d**) Recession type and (**e**) Jaw type. Recession depth was measured digitally between the most apical point of the gingival margin and the CEJ. Mean root coverage was calculated by dividing the difference between the preoperative and postoperative recession depth by the preoperative recession depth multiplied by 100 (Cairo et al. 2009). Complete root coverage was calculated by dividing the number of teeth with a postoperative completely covered root (root covered to or above the CEJ) by the total number of teeth included multiplied by 100 [30]

Mean root coverage was 65.06 ± 48.26% (maxilla: 68.84 ± 53.77%; mandible: 61.80 ± 43.31%. Differentiated by recession type, the mean root coverage was 61.47 ± 56.83% for RT1 and 68.32 ± 39.33% for RT2 (Fig. 4 (a)-(c)). Thus, neither recession type (*p* = 0.517) nor jaw type (*p* = 0.509) demonstrated an influence on mean root coverage.

When analyzed by tooth type, the mean root coverage values were as follows: 58.61 ± 42.94% for incisors, 82.22 ± 36.66% for canines, 74.75 ± 47.37% for premolars, and 44.31 ± 59.13% for molars. Although canines had a significantly higher mean root coverage than molars in the maxilla (*p* = 0.014), the overall mean root coverage was not influenced by tooth type (*p* = 0.053).

### Complete root coverage

Complete root coverage was achieved in 21 out of 82 teeth (25.61%) (maxilla: 28.95%; mandible: 22.73%). Differentiated by recession type, complete root coverage was accomplished in 28.21% for RT1 and 23.26% for RT2 (Fig. 4 (d), (e)). Thus, neither recession type (*p* = 0.450) nor jaw type (*p* = 0.696) had an influence on the ability to achieve complete root coverage. A statistical evaluation of the influence of tooth type on complete root coverage could not be performed.

### Mean recession reduction

**Fig. 5:**
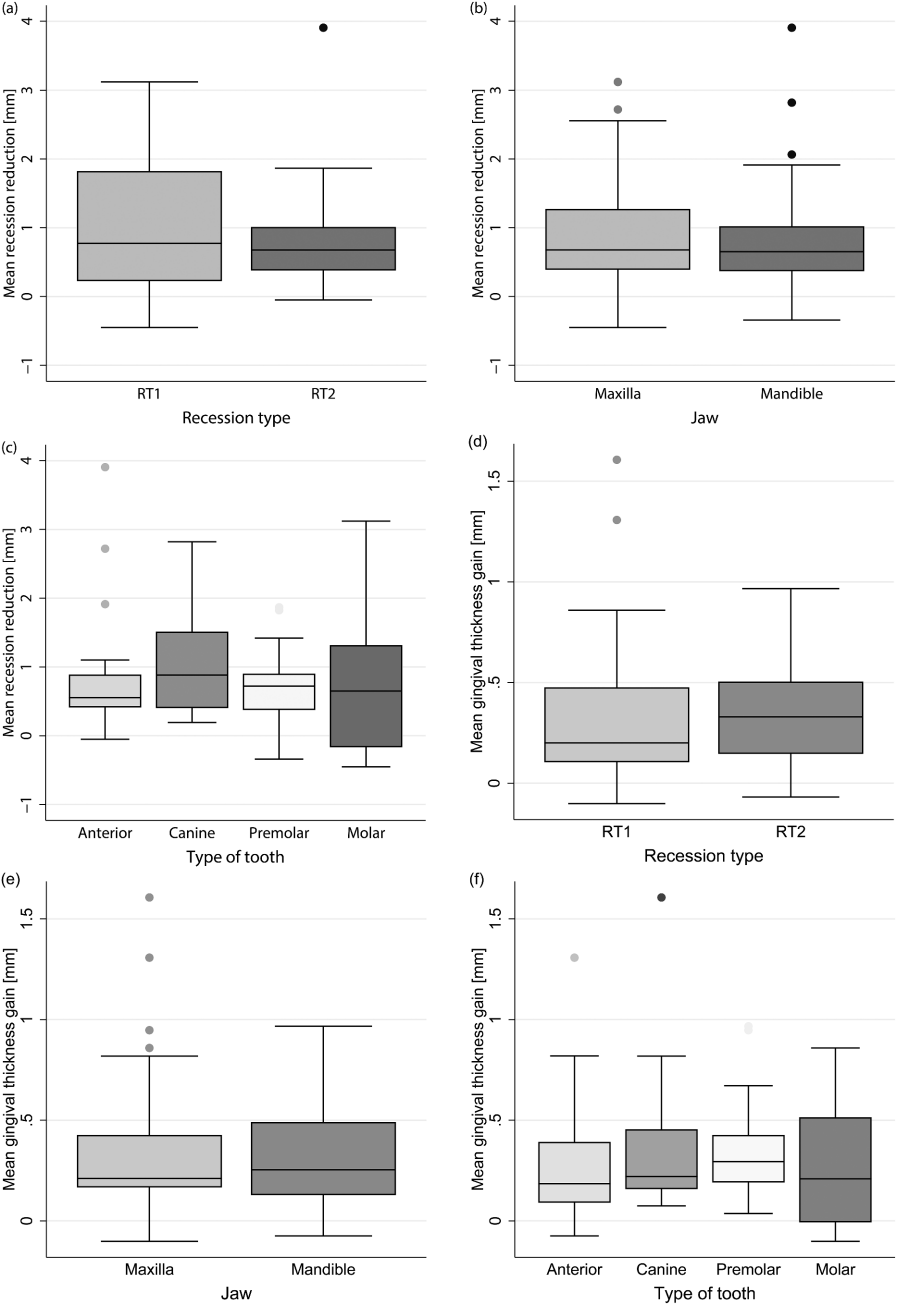
Mean recession reduction in mm according to (**a**) Recession type, (**b**) Jaw type and (**c**) Tooth type and mean gingival thickness gain in mm according to (**d**) Recession type, (**e**) Jaw type, and (**f**) Tooth type. Mean recession reduction was measured digitally and automatically by the GOM Inspect software as the difference between the most apical point of the preoperative and postoperative gingival margin or in the case of mean gingival thickness gain as the difference between the preoperative and postoperative gingiva at the most apical point of buccal recession

The mean postoperative reduction of recession was 0.87 ± 0.83 mm (maxilla: 0.88 ± 0.89 mm; mandible: 0.85 ± 0.77 mm). Differentiated by recession type, the mean recession reduction was 0.96 ± 1.00 mm for RT1 and 0.77 ± 0.63 mm for RT2 (Fig. 5 (a) - (c)). Thus, neither recession type (*p* = 0.286) nor jaw type (*p* = 0.814) demonstrated an influence on the mean reduction of the recession.

When analyzed by tooth type, the mean recession reduction was 0.88 ± 0.92 mm for anterior teeth, 1.10 ± 0.82 mm for canines, 0.72 ± 0.53 mm for premolars, and 0.86 ± 1.07 mm for molars. Tooth type (*p* = 0.544) had no significant effect on postoperative recession reduction.

### Gingival thickness

Due to impression artifacts in 10% of the cases, we were only able to measure soft tissue thickness in the median tooth axis at the most apical point of buccal recession. The mean gain of gingival thickness was 0.33 ± 0.30 mm (maxilla: 0.34 ± 0.36 mm; mandible: 0.32 ± 0.24 mm). Differentiated according to recession type, the gingival thickness gain for RT1 was 0.32 ± 0.37 mm and for RT2 0.34 ± 0.24 mm (Fig. 5 (d), (e)).

Thus, neither recession type (*p* = 0.811) nor jaw type (*p* = 0.708) demonstrated an influence on postoperative thickness changes. Differentiated by tooth type, the gain of gingival thickness was 0.30 ± 0.32 mm for anterior teeth, 0.37 ± 0.38 mm for canines, 0.35 ± 0.24 mm for premolars, and 0.29 ± 0.30 mm for molars (Fig. 5 (f)). Thus, tooth type (*p* = 0.534) showed no influence on gingival thickness changes.

### Intrarater and interrater reproducibility

Considering all measured parameters and all included teeth, the overall intrarater and interrater reproducibility of measurements in the present study was excellent, with an intraclass correlation coefficient of 0.954–0.999 and 0.937–0.999, respectively [33].

## Discussion

The limitations of conventional clinical assessment of gingival root coverage highlight the need for CEJ-independent and digital assessment methods. The digital evaluation workflow used in the present study provides an approach to assess gingival recession coverage outcomes, independent of the cemento-enamel junction (CEJ), and enables quantitative comparability. The modified coronally advanced tunnel technique (MCAT) in conjunction with the porcine dermal matrix (PDM) yielded encouraging results in terms of root coverage.

The CEJ is not a reliable reference value for soft tissue measurements, however it is the most commonly used. When opting for the CEJ, in spite of the inherent limitations, the overall rate for mRC and cRC in the present study is consistent with the reported range of 53–93% and 20–33% in previous RCTs using other porcine dermal matrices to cover Miller class I and II recessions using the modified coronally advanced tunnel technique [16, 20, 22], . There is only one RCT investigating Miller class III recessions, reporting a mRC of 83% and a cRCof 38% within a healing time of 12 months [32]. Thus, the recession coverage rate for PDMs is comparable to porcine collagen matrices (mRC: 71–91% and cRC: 14–22 [10, 34] and allogenic dermal matrices (mRC: 66–85%; cRC: 27–83% [35–37]) in available RCTs at 6–12 months postoperatively. However, especially for the coverage of multiple recessions, autologous connective tissue grafting remains the gold standard treatment due to its better predictability and long-term stability [38].

The inaccurate detection of soft tissue recessions relying on the CEJ is intensified by the most common clinical measurement method using a periodontal probe, which measures to the nearest 0.5 mm. Due to rounding errors, variations in measurement angle and position, this method results in deviations of up to 2.7 mm between individual measurements [23]. These factors contribute to measurement results which lack reliability and comparability across studies.

Much more accurate measurement results can be achieved using digital measurement methods compared to using a periodontal probe [39]. The measurements can be taken with an accuracy of 0.01 mm, demonstrating high inter- and intra-rater reliability [40].

In order to overcome the inaccuracy associated with the CEJ, the present study used a direct measurement of recession reduction between the pre- and postoperative marginal gingiva, leading to a recession reduction of 0.87 ± 0.83 mm (maxilla: 0.88 ± 0.89 mm; mandible: 0.85 ± 0.77 mm). Neither recession type (*p* = 0.286) nor jaw type (*p* = 0.814) demonstrated an influence on the mean reduction of the recession. To the best of the authors’ knowledge, this measurement approach has only been tested in vitro recently [40]. In contrast to the current approach, which uses the full arch to superimpose the preoperative and postoperative scans, Dritsas and colleagues limited the region of interest to the teeth immediately adjacent to the tooth being examined [40]. The narrowed region of interest used in that study allows scan superimposition without interfering with the measurement, even if new prosthetic restorations were performed on the remaining teeth (except the neighbooring teeth) during the follow-up period. The accuracy of scan superimposition appears to be comparable between matching the entire model arch (0.008 mm) and matching only individual adjacent tooth crowns (0.009 mm) [40]. There was no difference in accuracy, whether the deepest point of the marginal gingival margin was selected manually or automatically for the digital measurement. Therefore, it can be concluded that the gingival margin is an optimal measurement landmark due to its clear visibility and should be utilized in future studies. The STL files must be available in the same coordinate system to ensure the comparability of individual measurements; hence, the same scanner must be used throughout the entire study.

The average gingival thickness gain in the present study was 0.33 mm. Thus, our results are comparable to the current literature, which reports a gain in gingival thickness of 0.27–0.4 mm within an observation period of 12 months for recession coverage using porcine and allogenic dermal matrices [16, 22, 41]. However, autologous connective tissue grafting results in higher gain of gingival thickness of 1–1.96 mm [16, 22, 41]. In literature, soft tissue thickness measurements are frequently performed transgingivally with an endodontic file [22, 41]. The resulting measurement accuracy of approximately 0.5 mm [42] is very coarse for the measurement space in question and thus, apart from the invasiveness, rather speaks in favor of non-invasive and more precise digital measurement methods [43].

At this moment, making comparisons to other digital workflows is very challenging, as literature employs heterogeneous landmarks, various scan systems and software, or cemento-enamel junction (CEJ)-dependent measurements [26, 27, 44].

In the current study, the location of root coverage (maxilla vs. mandible) did not influence the root coverage rate. In contrast, previous studies evaluating root coverage rates have reported lower recession coverage rates for teeth in the mandible compared to teeth in the maxilla [45–47]. Possible explanations include reduced vascularization and dimensional stability due to the narrower papillae, as well as the reduced vestibular depth, which impedes coronal soft tissue mobilization in the mandible [45–47]. Chambrone and Chambrone utilized a periodontal dressing to reduce lip tension, which has also been discussed as a factor influencing root coverage [46]. However, none of the aforementioned studies employed extraoral taping to reduce postoperative facial mimic muscle activity, which might have contributed to the results of the current study. Although tooth type has been described as one of the factors influencing the long-term outcome of surgical root coverage [45], tooth type was not associated with root coverage rate in this study. A few studies described a higher root coverage rate for anterior teeth, possibly due to better accessibility of the anterior region [7, 48] these results, however, were not significant. In addition to differences in recession etiology, differences in root coverage outcomes may depend on the surgical difficulty of covering single monolateral or multiple bilateral recessions [49].

Certain limitations of this study should also be acknowledged. Due to minimal color differences, the CEJ was hard to discern on the digital models. Consequently the plaster cast had to serve as reference, which is as the clinical assessment, suboptimal for CEJ detection, as highlighted by Zuchelli et al. [24]. Additionally, due to impression artefacts (10% o all cases), particularly in the gingival papillae region, only the thickness along the median tooth axis at the most apical point of the buccal recession could be assessed. Due to these challenges, it becomes evident that a fully digital workflow incorporating digital scanners and consistent, universal landmarks independent of the CEJ across all studies is necessary.

In summary, the digital measurement technique employed offers an approach to evaluate gingival recession coverage independent of the CEJ. We present the clinical outcome of root coverage using a PDM after 6 months, regardless of the type of recession, tooth type, and jaw type. Digital, reproducible and universal landmarks replacing the CEJ need to be defined in the future for digital root coverage assessment to allow comparability between different studies and to increase accuracy. However, whether the present method alone provides sufficient information to assess the coverage success will need to be clarified through further studies. A reliable evaluation of the long-term success of recession coverage across studies, will contribute to the development of new surgical techniques and materials.

## Conclusions

The present findings indicate that the digital measurement workflow used provides an approach to evaluate gingival recession coverage outcomes independent of the CEJ. The PDM used in combination with MCAT yielded promising results for root coverage at 6 months.

## Data Availability

The datasets used in the present study are available from the corresponding author upon reasonable request.

## Abbreviations

CEJ: Cementum-enamel junction
cRC: Complete root coverage
mRC: Mean root coverage
mRR: Mean recession reduction
MC: Miller class
MCAT: Modified coronally advanced tunnel
PDM: Porcine dermal matrix
RCT: Randomized Clinical Trial
RT: Recession type
STL: Stereolithography
